# The role of gravitational effects and pre-puncture techniques in reducing pneumothorax during CT-guided lung biopsies

**DOI:** 10.1007/s11547-025-02007-w

**Published:** 2025-04-15

**Authors:** Michael P. Brönnimann, Maria C. Barroso, Leonie Manser, Bernhard Gebauer, Timo A. Auer, Federico Collettini, Patrick Dorn, Adrian T. Huber, Johannes T. Heverhagen, Martin H. Maurer

**Affiliations:** 1 Department of Diagnostic, Interventional and Pediatric Radiology, Inselspital, Bern University Hospital, University of Bern, Rosenbühlgasse 27, 3010 Bern, Switzerland; 2 Department of Radiology, Charité-Universitätsmedizin Berlin, Augustenburger Platz 1, 13353 Berlin, Germany; 3 Clinician Scientist Program, Berlin Institute of Health at Charité-Universitätsmedizin Berlin, Berlin, Germany; 4 Department of Thoracic Surgery, Inselspital, Bern University Hospital, University of Bern, Freiburgstrasse 4, 3010 Bern, Switzerland; 5 Department of Radiology and Nuclear Medicine, Lucerne Cantonal Hospital, University of Lucerne, Lucerne, Switzerland; 6 Department of Radiology, Carl Von Ossietzky University Oldenburg, Oldenburg, Germany

**Keywords:** Pneumothorax, Biopsy, Image-guided biopsy, Postoperative complications tomography, Lung, Patient positioning

## Abstract

**Purpose:**

The study aimed to evaluate whether the relative height (RH) of the entry point (EP) during CT-guided lung biopsies, adjusted for patient positioning, can predict the risk of pneumothorax during the intervention, leveraging the gravitational effects on pleural pressure.

**Materials and methods:**

We retrospectively analyzed 128 percutaneous CT-guided lung biopsies performed at a single center between January 2018 and December 2023. Patients were grouped based on pneumothorax occurrence. Various measurement methods indirectly assessed the influence of gravitational force on pleural pressure, focusing on the RH at the EP with prone positioning adjustments (PP). Other confounding factors like patient demographics, lesion characteristics, pre-puncture fluid administration and other procedural details were assessed. Test performance metrics were compared using Chi-Square, Fisher’s exact, and Mann–Whitney U tests. Univariate and binomial logistic regression assessed the influence of different parameters on pneumothorax occurrence.

**Results:**

All measurements of lower RH at EP and pre-puncture fluid administration were significantly associated with a reduced incidence of peri-interventional pneumothorax (*p* < 0.01). The RH at EP adjusted for the prone position demonstrated the best predictive performance (AUC = 0.844). After adjusting for various confounding factors, both lower RH at EP adjusted for the prone position (OR 110.114, *p* < 0.001) and pleural fluid administration (OR 0.011, *p* = 0.011) remained independently associated with a lower risk of pneumothorax.

**Conclusion:**

Strategic use of gravity by selecting the lowest possible entry point, ideally positioning the patient laterally, combined with pre-puncture pleural fluid administration, could be the key to reducing pneumothorax in CT-guided lung biopsies.

## Introduction

Percutaneous CT-guided lung biopsy (CTLB) is a well-established, minimally invasive procedure widely used for the histological evaluation of focal lung lesions. With the increasing implementation of lung cancer screening and the growing use of CT imaging, the detection rate of pulmonary masses has risen significantly. Since definitive management often depends on histopathological confirmation, CTLB plays a crucial role in clinical decision-making [1]. This technique has demonstrated high diagnostic accuracy, ranging from 83 to 97%, making it a reliable tool for tissue characterization [2]. Moreover, CT-guided percutaneous transthoracic needle biopsy remains one of the most effective methods for diagnosing thoracic masses, offering both precision and safety [3].

Pneumothorax occurs when air enters the pleural space due to alveolar wall rupture or thoracic trauma, also in the context of lung biopsies, causing increased pleural pressure and lung collapse [4]. Pneumothorax is the most common complication of CT-guided lung biopsies, with incidence rates ranging from 8 to 69%, depending on study parameters and patient populations [4–18]. While most pneumothoraces are minor and asymptomatic, and resolve spontaneously, larger forms occur in 5–15% of patients, requiring drainage, additional imaging, and often hospital admission. These interventions can increase healthcare costs by 300–400% compared to biopsies without complications [10, 13, 16, 19–25].

While various post-procedural techniques have been studied to reduce the risk of pneumothorax (e.g., [12, 14, 22, 26–30]), pre-procedural approaches have not been thoroughly explored. One strategy involves positioning the patient in a specific way during or after the biopsy, but results have been inconsistent. Performing the biopsy with the patient in a biopsy-side down position has generally not reduced pneumothorax risk [22, 31]. The so called PEARL approach suggests that this positioning may offer protective benefits [32, 33]. Additionally, evidence suggests that pre-procedural patient positioning and pre-biopsy fluid administration can further minimize the risk of pneumothorax [34].

**Fig. 1:**
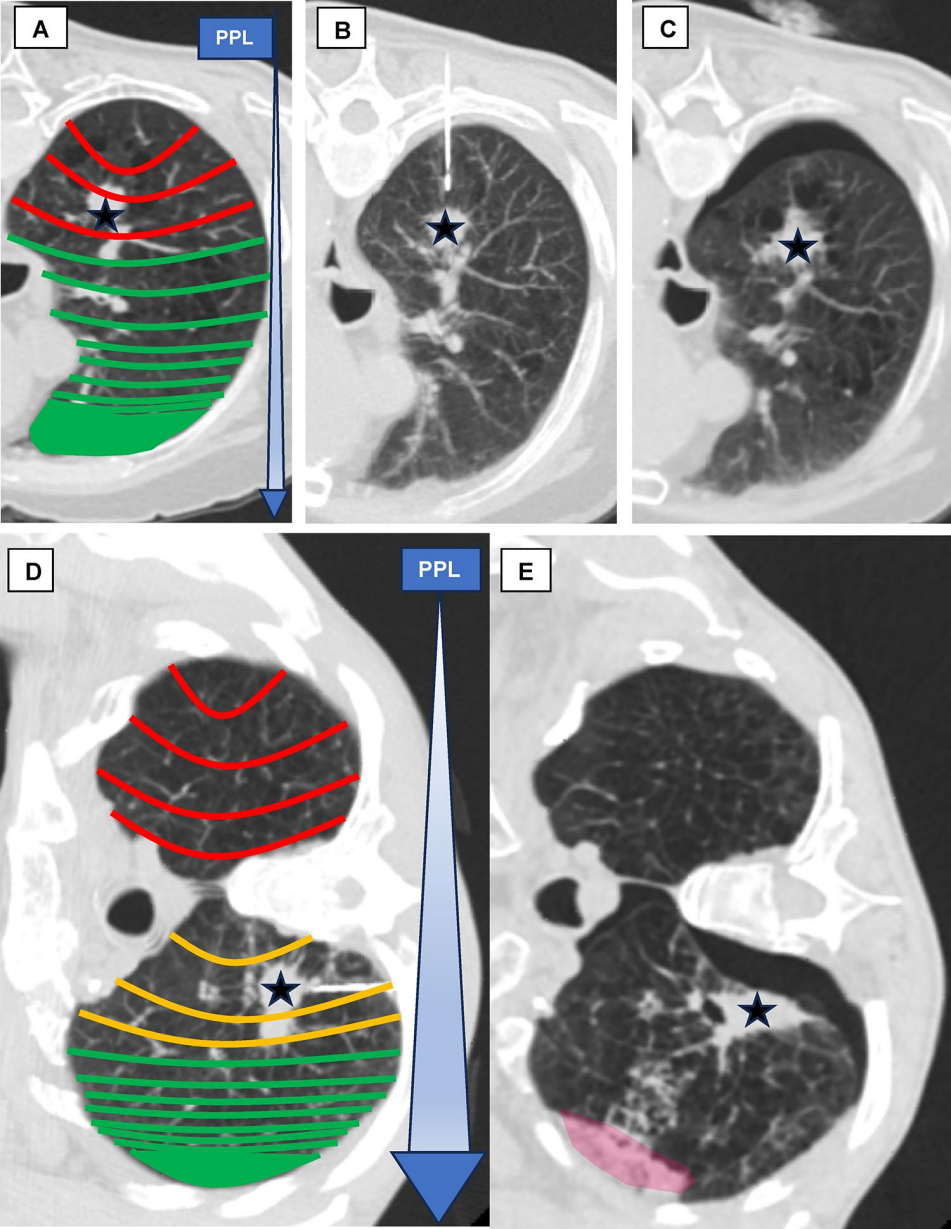
Graphical abstract—schematic illustration of the presumed influence of gravitational force (GF) based on patient position In This Study. **A**, **D** Pleural pressure (PPL) decreases linearly from the non-dependent upper to the dependent lower lung regions, primarily due to the gravitational effects of lung weight [35, 39, 40, 43–47]. Represented by red, orange and green gravitational field lines to indicate the degree of gravity. The blue arrow indicates the pleural pressure that steadily increases due to gravitational force. **B, C** The patient was in the prone position with the coaxial needle inserted posteriorly, targeting the lesion (asterisk). A pneumothorax occurred immediately post-intervention. **D** The patient was positioned in the lateral decubitus position with the biopsy-side down to prevent pneumothorax. The coaxial needle tip is near the target lesion (asterisk). **E** Following a full core lung biopsy, a pneumothorax and a pulmonary hemorrhage (red area) were observed

These discrepancies in study outcomes may be due to the fact that pleural pressure varies throughout the pleural cavity. Negative pleural pressure is more pronounced in the upper, non-dependent lung regions than in the lower, dependent areas. This difference arises from the lung’s weight and possible shape discrepancies between the lung and rib cage [35, 36]. The lung’s weight primarily influences this gradient, creating a linear, vertical gravitational gradient of less than 1 cmH_2_O/cm [37–40]. Pleural pressure also varies with body position [41]. Animal models have shown that the gradient is approximately half as large in the prone position compared to the supine or lateral positions [42]. Given this, we hypothesized that the linear effect of gravitational force on pleural pressure could be leveraged to reduce pneumothorax risk. Specifically, targeting patient positioning to select the lowest possible entry point into the pleura may help minimize the risk. This study aimed to investigate whether the relative height of the entry point during CT-guided lung biopsies could predict the risk of pneumothorax (Fig. 1).

## Material and methods

### Study population

**Fig. 2:**
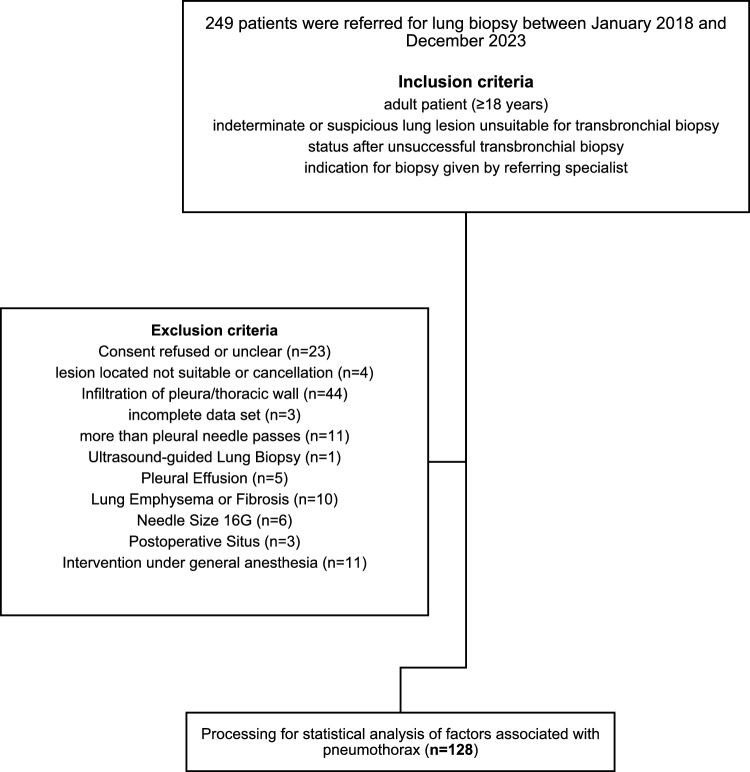
Flowchart with the study population

This study retrospectively analyzed 249 percutaneous CT-guided lung biopsies performed at a Swiss university hospital between January 2018 and December 2023. Exclusion criteria were carefully defined to reduce the risk of confounding factors that could distort the study results. Pleural effusion, for example, is known to raise pleural pressure, which may influence measurements or outcomes related to pleural cavity physiology [4, 34]. Similarly, pathological lung conditions such as emphysema and fibrosis can lead to abrupt and significant changes in pleural pressure, which could undermine the consistency and reliability of results [37, 39, 48, 49]. Biopsies performed with a 16-gauge (G) needle were excluded due to a small sample size that could lead to unreliable results. Additionally, cases involving more than one pleural passage were excluded due to the well-established risk of pneumothorax [6] which could introduce additional complications and affect post-procedural outcomes. Interventions performed under general anesthesia were excluded. General anesthesia impacts ventilation and, consequently, pleural pressure, while the varying modes of ventilation were not systematically recorded. This introduces a risk of confounding, as changes in pleural pressure could be attributed to anesthesia and ventilation differences rather than the procedure itself, thereby complicating the interpretation of the results [50] (Fig. 2).

### Baseline evaluation and biopsy technique

**Fig. 3:**
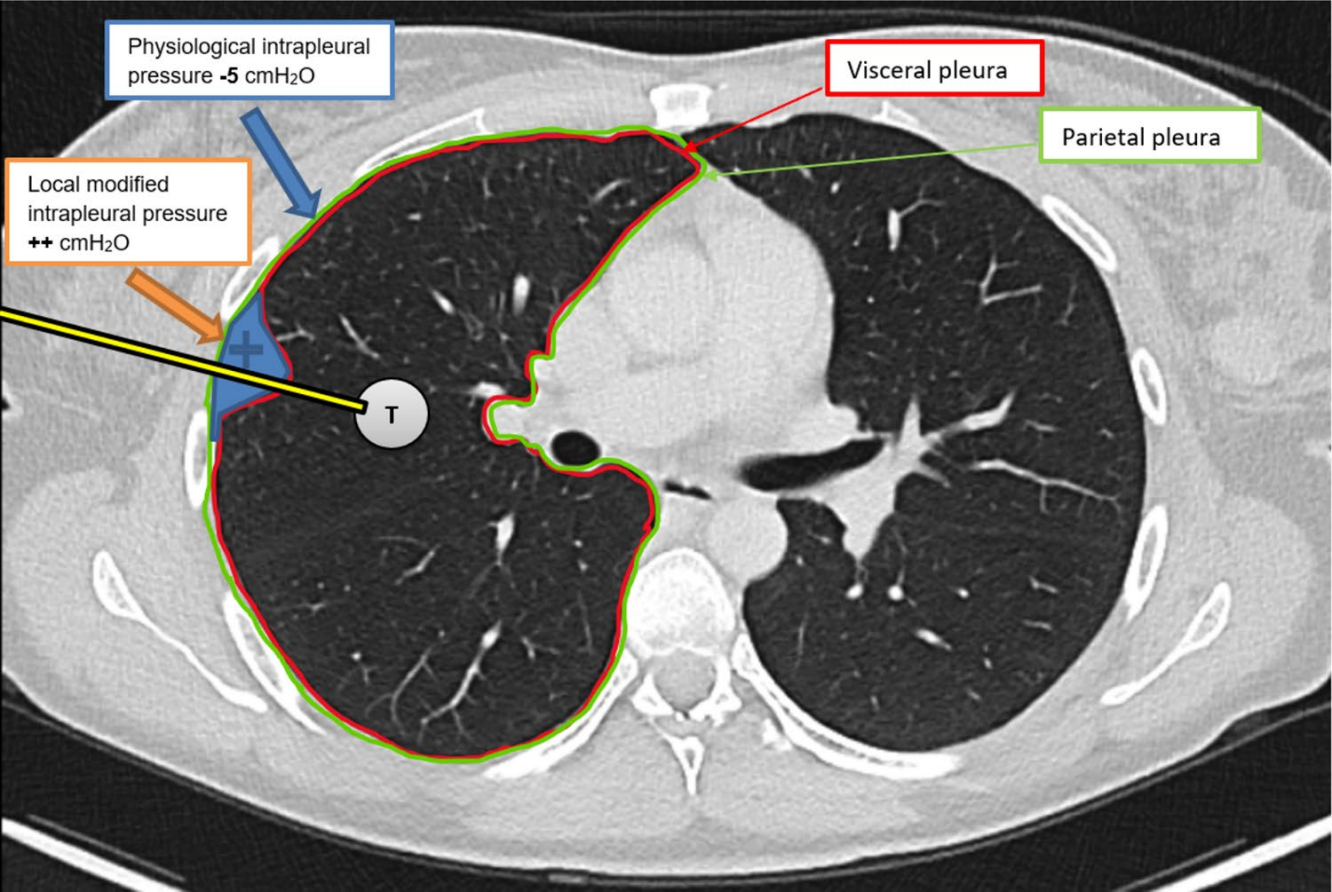
A schematic illustration of CT-guided lung biopsy with localized fluid application in the subpleural space to elevate pleural pressure, thereby reducing the risk of pneumothorax

All patients underwent a baseline clinical evaluation, including a review of their medical history and standard blood tests. Procedure requirements included an International Normalized Ratio (INR) value below 1.5 or a Quick value above 60%, a Hemoglobin (Hb) level over 80 g/L, and a platelet count exceeding 50 × 10^9/L, with blood test results no older than 5 days. Non-steroidal anti-inflammatory Drugs (NSAIDs) and clopidogrel were discontinued 5 days prior, heparin 6 h before, rivaroxaban 1 day before, and both dabigatran and edoxaban 3 days prior to the intervention, in accordance with established guidelines. If the biopsy posed a significant risk during normal breathing (patient-specific conditions or lesion too close to vital structures), it was rescheduled under anesthesia with jet ventilation. Four experienced interventional radiologists, each with 7 to over 10 years of experience, performed all lung biopsies. The procedures were CT-guided using a Toshiba Asteion 4SL scanner, with a 17- or 19-gauge coaxial needle and an 18- or 20-gauge semiautomated biopsy system (SemiCut side-cutting for 18-gauge; Medical Devices Lease S.A., Zug, Switzerland, or CorVocetTM full core; Merit Medical Systems, Utah, United States). Biopsy planning was based on a non-contrast chest CT with 1 mm reconstruction increments, adhering to the gold standard for needle path planning. Special care was taken to avoid pulmonary vessels, and crossing pulmonary fissures was strictly avoided. The Interventional radiologist (IR) selected patient positioning based on experience and the patient’s capabilities. Local anesthesia (1% lidocaine, max 20 mL) was administered. Breathing instructions were omitted to prevent hyperventilation, which could prolong the procedure. After tissue sampling, the needle was promptly withdrawn without a sealing agent. When the IR opted for fluid administration, a 10 mL depot of 1% lidocaine was placed subpleurally or within the pleural cavity to increase pleural pressure. Positioned in the soft tissue near the parietal pleura, this depot avoided saline to minimize complications from needle manipulation (Fig. 3). A 20-gauge needle was used, with fluid continuously administered during local anesthesia without needle changes. The needle’s position was carefully monitored by successive CT scans to avoid penetrating the visceral pleura. If diffusion was inadequate, the needle was repositioned, and the fluid readministered. The puncture needle was then replaced with the coaxial needle for biopsy. A follow-up CT scan was performed immediately after needle withdrawal and again five minutes later. If a progressive pneumothorax was detected on either scan, a drainage system (Safe-T-Centesis TM 6 or 8 French (F)) was employed. Patients were then monitored on the ward for routine vital signs for four hours post-procedure. If no complications arose during monitoring, patients were discharged home. In cases where the control CT scan revealed a non-progressive pneumothorax, a chest X-ray was performed six hours later for further assessment. The patient was admitted for overnight observation if the pneumothorax exceeded 2 cm apically.

### Physical considerations for indirectly measuring the impact of gravitational force on pleural pressure

Measurements were taken in the CT slice where the biopsy needle hit the target to capture the linear vertical gravitational gradient of pleural pressure. This approach was chosen to accurately reflect the impact of gravity on pleural pressure [35, 37, 38, 43–45, 51]. Due to this linearity, the different heights of the entry points should correlate accordingly. To account for varying thorax sizes, which were mostly gender-specific and dependent on body size [52], the absolute measured values are adjusted relative to the maximum anteroposterior diameter. In the lateral position, the total lung height was used as a reference, as it is known that the weight of the mediastinal structures and the non-dependent lung exerts additional force on the dependent lung [38, 46]. In the axial slice where the lesion was reached with the coaxial needle, two distances were measured in millimeters. First, the maximum anteroposterior distance (APD) was determined to represent the height-dependent, linear increase in pleural pressure. This was based on our hypothesis that at the most cranial point, the protective effect of lung self-weight on pleural pressure is the smallest, resulting in the highest risk of pneumothorax.

**Fig. 4:**
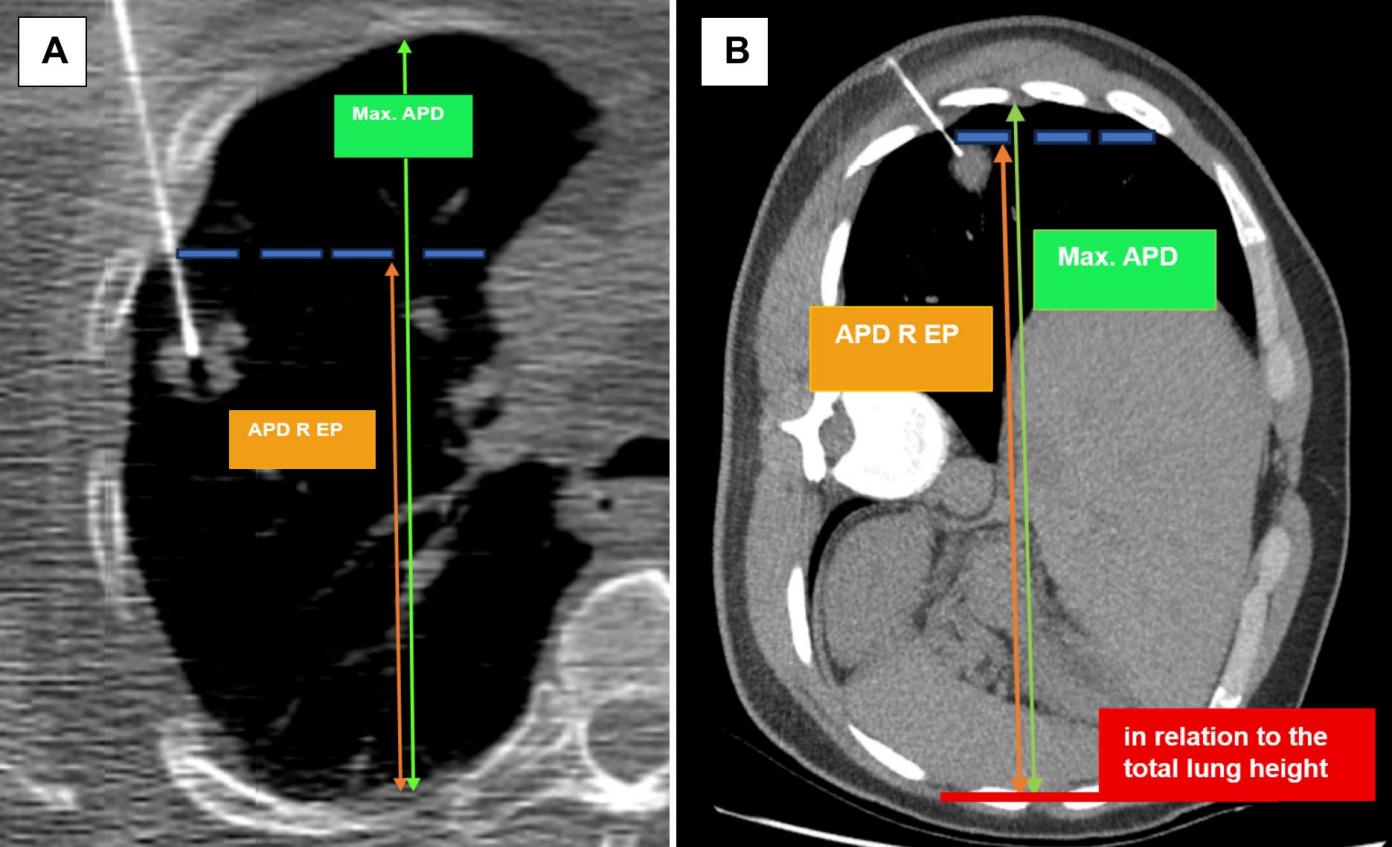
Indirect measurement of the gravitational force influence, derived from the vertical pleural pressure gradient (PPL) at the entry point. **A** Patient in the supine position. The suspicious lung mass is accessed with the biopsy needle from the anterior. The entry point height measured in millimeters was correlated with the corresponding lung height at the same layer level and on the same side. **B** In the lateral position, the total lung height is used as a reference. The indirect GF, representing RH for EP without adjustment for PP, was calculated as follows: APD R EP/ max. APD. Max. APD, maximum anteroposterior distance; APD R EP, anteroposterior distance in relation to the entry point; GF, gravitational force; RH, relative height; EP, entry point; PP, prone position all distances are measured in millimeters (mm)

**Fig. 5:**
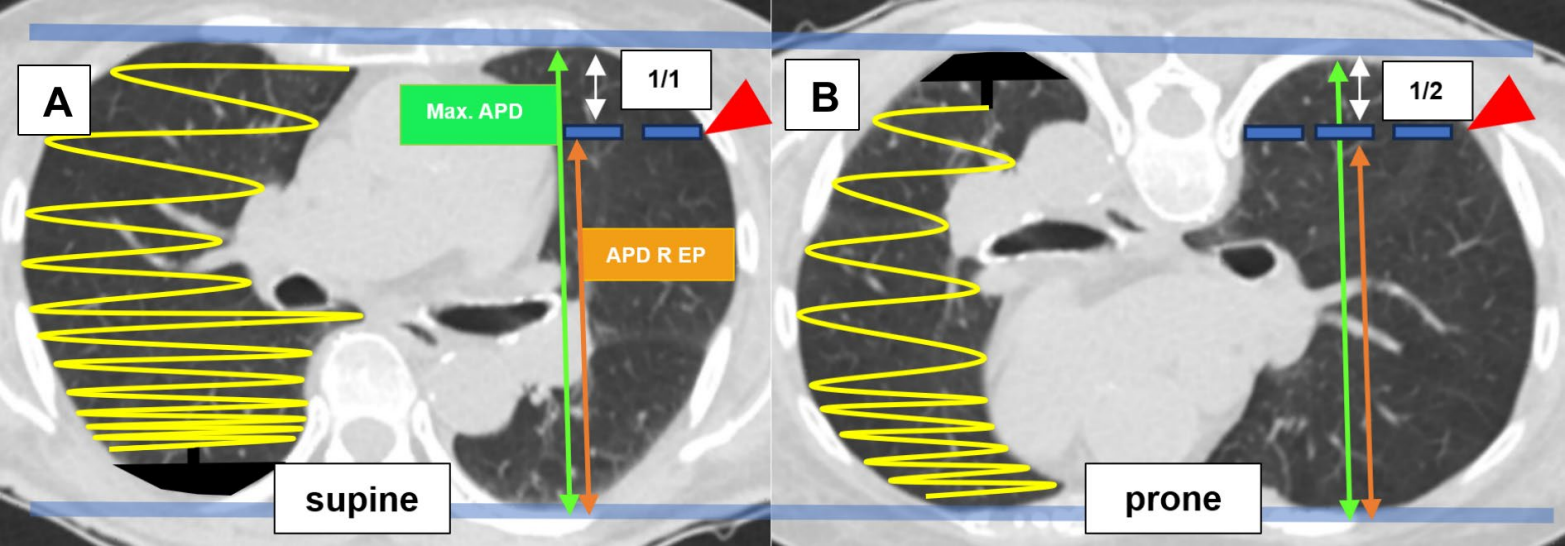
Indirect measurement of the influence of gravitational force, derived from the vertical pleural pressure gradient (PPL) at the entry point, accounting for the specific conditions in the prone position. Same patient in **A** supine and **B** prone position with puncture site (red arrowhead) at the same level in the upper third of the left thorax. According to Kallet’s model [41], in the supine position, gravity increases strain due to the Slinky® effect of a triangular spring suspended from its apex (supine position). In contrast, in the prone position results in more even strain distribution and reducing stress inhomogeneity. This could explain the halved vertical pleural pressure gradient in the prone position observed in the dog model of Hoppin et al. [42]. **B** Consequently, the indirect GF as RH at EP with adaptation for PP was calculated in the prone position as follows: (APD R EP/ max. APD)/2. The double arrow indicates the difference to the maximum APD. Max. APD, max. anteroposterior distance; APD R EP, anteroposterior distance in relation to the entry point; GF, gravitational force; RH, relative height; EP, entry point; PP, prone position

In the second step, the entry point of the coaxial needle was identified. A perpendicular line to the maximum APD was drawn, and the corresponding second height was measured. This height was then expressed as a ratio relative to the total maximum APD. According to our hypothesis, the lower the entry point height relative to the total maximum APD, the higher the protective pleural pressure (Fig. 4)**.** A further measurement method, relative height (RH) at the entry point (EP) with adaption for the prone position (PP), was employed to capture the suspected specific conditions in the prone position. According to Kallet’s model [41], when a person is in the supine position, gravity amplifies strain on the lung due to the "Slinky effect," analogous to a triangular spring suspended from its apex. This configuration leads to uneven strain distribution across the lung. Conversely, the strain distribution becomes more uniform in the prone position, reducing stress heterogeneity. This effect may account for the observed reduction in the vertical pleural pressure gradient by half in the prone position, as demonstrated in the dog model by Hoppin et al. [42]. The more even strain distribution in the prone position could result in a more balanced pleural pressure gradient, potentially mitigating localized stress and improving overall lung function. In simple terms, when lying on the back, gravity causes the lung tissue to stretch unevenly, like a spring hanging from a single point, leading to higher pressure differences. However, when lying on the stomach, the lung is more evenly supported, reducing these pressure differences. Considering the assumption that the gradient in the prone position is only half as large, we adjusted our calculated quotient accordingly by dividing it by two in this position (Fig. 5).

**Fig. 6:**
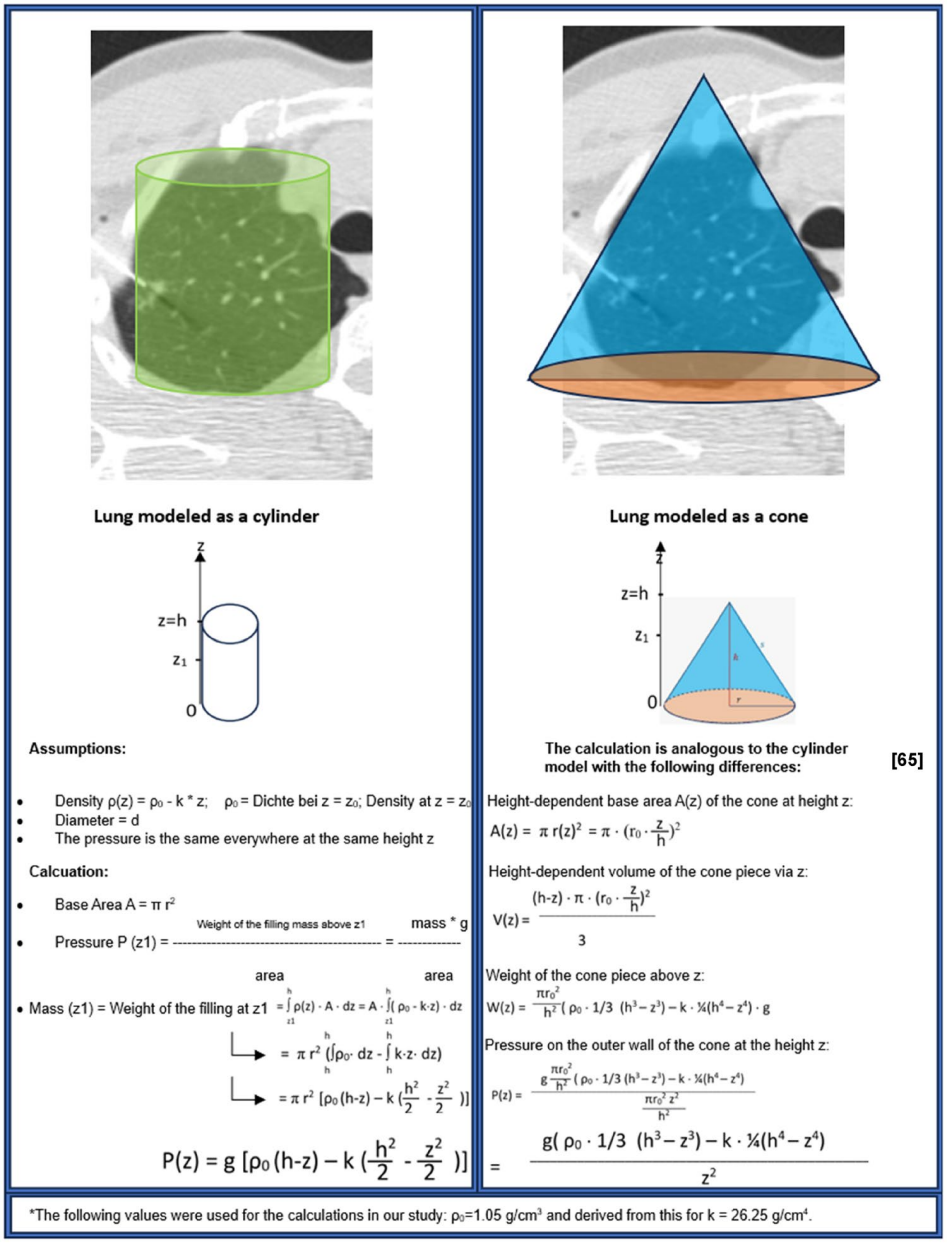
Physically approximated models derived from geometric shapes to assess the effect of gravitational force on pleural pressure

Physical calculations were performed using two geometric shapes, a cylinder and a cone, which best approximate the lungs in axial planes. This approach aimed to model and mathematically quantify the effect of gravitational force on pleural pressure at biopsy height based on lung shape. This analysis led to the conclusion that regardless of whether the lung is modeled as a cone or a cylinder, the pressure on the outer wall at the entry point height (z) is due to the gravitational force acting on the lung segment above z. Initially, this pressure decreases linearly as z increases. Based on the findings of Hopkins et al.[47], we assumed that lung density increases linearly with decreasing height z. Consequently, pressure is further reduced by a quadratic factor, which depends on the difference between the total height and the current height (Fig. 6). All measurements and formulas can be found in Figs. 4, 5 and 6 and were taken from the axial slice where the biopsy needle reached the lesion.

**Fig. 7:**
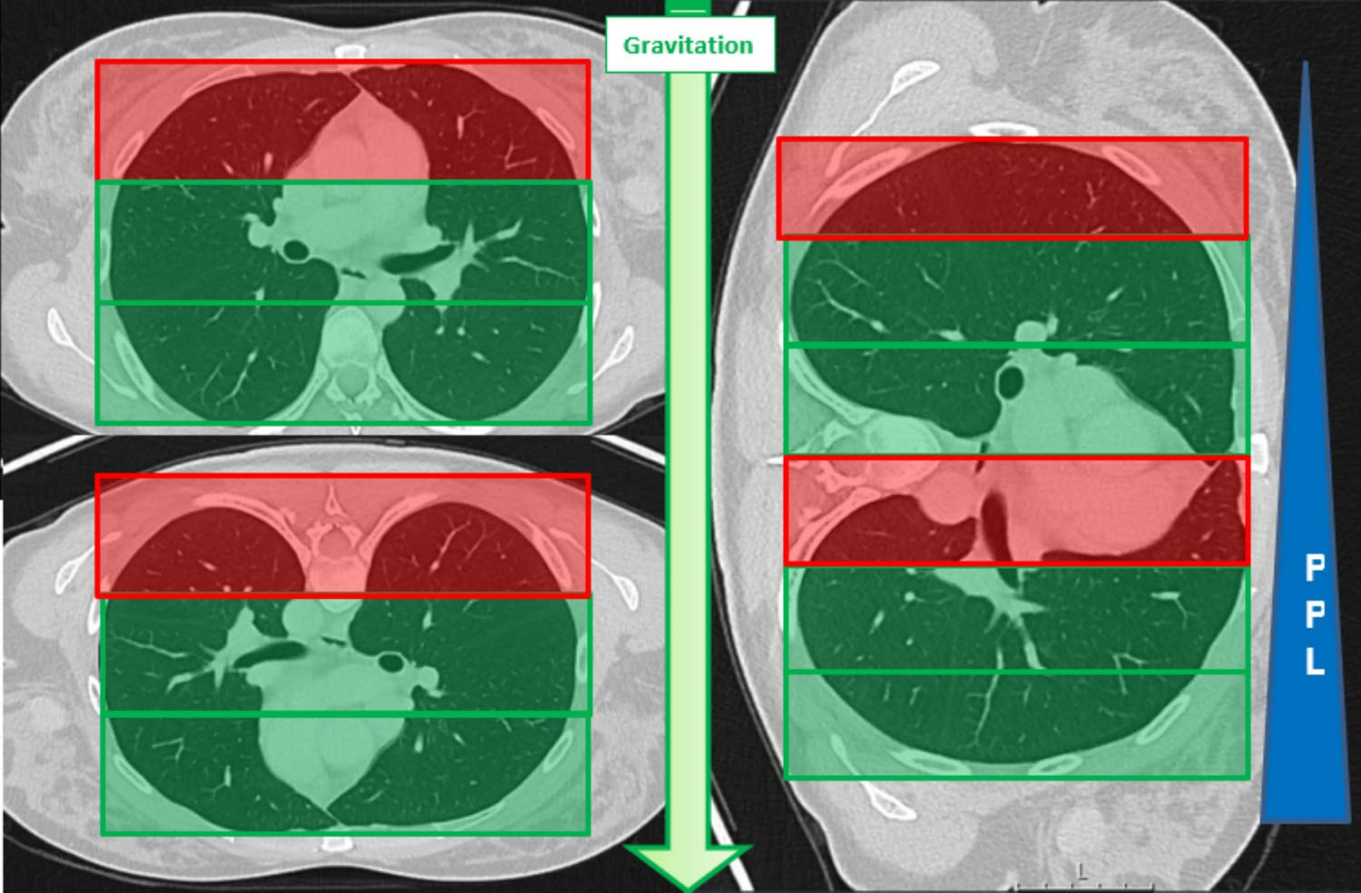
Schematic illustration of the zoning used for this study according to position-dependent gravitational effect on pleural pressure (PPL). For zoning, we applied the rule of thirds. Only the zone "RED" was determined as non-dependent

The final measurement method (qualitative) utilizes a simplified visual zoning system by dividing axial planning images into three equal sections and using non-anatomical landmarks for orientation. The red zone, representing the non-dependent lung area, is defined as the third where gravitational force is greatest. The remaining two-thirds are designated as the dependent lung area (Fig. 7). According to Stenqvist et al. [35], the schematic representation of gravitational effects on pleural pressure suggests that pleural pressure is considered positive from the middle third downward, attributed to progressive lung collapse. To simplify matters, the overall shape of the thorax and the determination of the lung’s center of gravity were not included in the calculations.

### Data collection

All procedures were evaluated by a board-certified interventional radiologist with nine years of experience and a radiology resident with three years of experience. Both reviewers were blinded to the patient’s medical history and did not participate in the interventions. The interventional images were analyzed using Sectra Workstation software (Model IDS7, Version 24.2, Patch 4/2022; Sectra AB, Linköping, Sweden). We recorded patient demographics, biopsy positioning, the maximum anteroposterior distance (APD) and the APD at the level of entry point, access route according to our zone classification, lesion size, lesion location, distances from skin to the lesion (along needle pathway), from skin to pleura and from pleura to lesion, fluid application in or near to the pleural space, biopsy angle, needle size, biopsy system, number of samples, procedure time (time difference in minutes from first CT image to first control CT image after biopsy), and the name of the IR performing the procedure. Histological results from the target lesion and the patient’s post-intervention history were retrospectively gathered from the electronic medical record. All metric measurements were in millimeters.

### Statistical analysis

All statistical analyses were performed using IBM SPSS Statistics for Windows, version 28 (IBM, Armonk, NY). Univariate analyses were performed using the Chi Square and Fisher’s Exact tests for categorical variables and the Mann–Whitney U test for continuous variables. All tests were two-sided, with a significance level set at *p* < 0.05. Normal distribution was assessed using the Kolmogorov–Smirnov test. The ideal measurement method should have a high area under the curve (AUC) value. We used Spearman’s correlation for continuous variables and contingency correlations for categorical variables to identify strong correlations between variables. The Phi coefficient was calculated for categorical variables, and the Pearson correlation coefficient was used for continuous variables to assess effect sizes. When variables were highly correlated, only the variable with the highest effect size was included in the logistic regression model. We employed binominal logistic regression to examine potential confounders and risk factors for pneumothorax and evaluated model fit with the Hosmer–Lemeshow test. To avoid overfitting, we followed the rule of ten by including up to five independent variables in the logistic regression model, based on their significance, while ensuring a minimum sample size of *n* ≥ 25 for categorical predictors.

## Results

### Study population

A total of 128 biopsies met the inclusion criteria, with a mean patient age of 65.4 ± 13.4 years (range 18–89 years), including 78 men (61%) and 50 women (39%). Of these biopsies, 62% of the lung nodules were malignant, predominantly metastases, with about one-third being primary lung tumors. The remaining 38% of nodules were benign. Non-usable results were observed in only 3% of the cases. Variables such as age, sex, patient position, lesion size, lesion location, number of samples, biopsy angle, procedure time and distances from skin-to-lesion/ pleura-to-lesion did not show significant differences between the groups (Table 1).

**Table 1:** Univariate analysis of patient demographics and interventional parameters

Survey of lung biopsies													
Parameter	All (*n* = 128)			No pneumothorax (*n* = 54)			Pneumothorax (*n* = 74)			*P* Value	φ	CC/SC	PCC
Female	50		39%	25		46%	25		34%	0.199			
Age (y)	65.39	±	13.40	62.74	±	15.60	67.32	±	11.25	0.090			
Lesion size (mm)	25.00	±	19.96	26.74	±	20.41	23.73	±	19.68	0.322			
Patient position										0.923			
Supine	51		40%	21		39%	30		41%				
LD	42		33%	17		31%	25		34%				
Prone	35		27%	16		30%	19		26%				
Lesion location										0.286			
UL	57		45%	21		39%	36		49%				
LL/ML/L	71		55%	33		61%	38		51%				
Indirect measurements of EGF													
RH at EP with adaption for PP	0.75	±	0.27	0.56	±	0.28	0.89	±	0.16	< 0.001*		< 0.001*	− ***0.5558***
RH at EP without adaption for PP	0.74	±	0.27	0.55	±	0.27	0.88	±	0.17	< 0.001*		< 0.001*	− ***0.5550***
PMC "cone"	− 14727067	±	5386321	− 3115370	±	8001672	− 195062	±	− 822814	< 0.001*			− ***0.5212***
PMC "cylinder"	− 1838028	±	2193693	− 9229247	±	2510728	584308	±	1148415	< 0.001*			− ***0.4997***
Access route ND lung region	61		48%	13		24%	54		73%	< 0.001*	**0.4840**		
Needle size										< 0.001*			
18 G	90		70%	28		52%	62		84%				
20 G	38		30%	26		48%	12		16%				
Biopsy system										< 0.001*			
Side-cut	63		49%	15		28%	48		65%				
Full-core	64		50%	39		72%	25		34%				
Number of samples (*n*)										0.577			
1 and 2	29		23%	10		19%	19		26%				
3	59		46%	25		46%	34		46%				
4	24		19%	12		22%	12		16%				
5	16		13%	7		13%	9		12%				
Pleural fluid application	29		23%	27		50%	2		3%	< 0.001*			
Biopsy angle (degree)	62.90	±	18.64	63.75	±	18.84	62.27	±	18.60	0.654			
Distance SL (mm)	62.48	±	22.66	67.00	±	24.06	59.18	±	21.14	0.05			
Distance PL (mm)	17.85	±	15.41	18.41	±	13.72	17.45	±	16.62	0.23			
Distance SP (mm)	44.63	±	15.77	48.59	±	17.98	41.74	±	13.33	0.037*			
Procedure time (min)	29.09	±	20.36	26.59	±	10.90	30.91	±	25.03	0.139			

The column of φ, CC/SC and PC shows the corresponding *P* values results. The tests that belong together are highlighted in italics, bold and bold italics.

Y, year; mm, millimeter; UL, upper lobe; LL, lower lobe; ML, middle lobe; L, lingula; EGF, effect of gravitational force; RH, relative height; EP, entry point; PMC, physical model calculation; ND, non-dependent; PP, prone position; G, gauge; min, minutes; *φ*, phi-coefficient; CC, contingency coefficient; SC, spearman correlation coefficient; PCC, Pearson correlation coefficient=*r*.

Unless stated otherwise, data are mean ± standard deviation.

X^2^ (R X 2), Fisher’s exact test and the Mann–Whitney U test were used to calculate the statistical difference between groups of categorical, dichotomous, and continuous variables, respectively. A value of φ = 0.1 is considered to be a small effect, 0.3 a medium effect, and 0.5 a large effect. The same applies to *r*, regardless of the sign [53].

^*^Statistically significant (defined *P* < 0.05).

### Pneumothorax after CT-guided lung biopsy

**Fig. 8:**
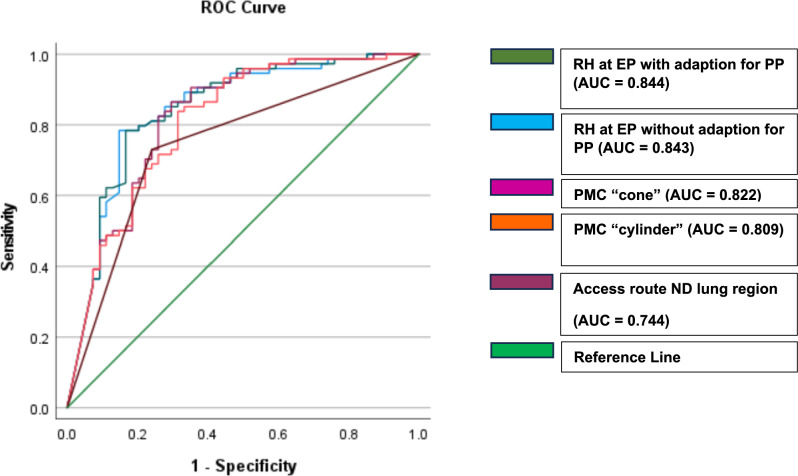
Validation of ROC (Receiver Operating Characteristic) Curves and Performance Metrics for Gravitational Force Parameters in Pneumothorax. RH, relative height; PP, prone position; PMC, physical measurement calculation; ND, non-dependent

Seventy-four patients (57.8%) developed a pneumothorax following the biopsy, with the highest incidence observed in the 55–69 year age group. In 7% of these cases, drainage was required, but no patients needed further intervention or surgery. The univariate analysis revealed that all indirect measurement methods used to assess the effect of gravitational force on pleural pressure showed a highly significant difference between the two groups (*p* < 0.01). Comparison of Pearson correlation coefficients and the Phi coefficient revealed that RH at EP with adaption for PP had the highest effect size in distinguishing cases with regard to pneumothorax occurrence in our cohort (*r* = − 0.5558; strong [53]). Additionally, pneumothoraces occurred significantly more frequently with the larger needle (18G) in 62 out of 74 cases (84% vs. 20G, 12/74; 16%, *p* < 0.01), the usage of the side-cut system in 48 out of 74 cases (65% vs. full core biopsy system with 25/74, 34%, *p* < 0.01), a smaller skin-to-pleura distance (*p *< 0.01), and when pre-puncture fluid administration was not performed (2 out of 72 cases, 3% vs. 27 out of 54 cases; 50%, *p* < 0.01). Performance metrics of measurement methods to predict pneumothorax showed that also RH at EP with adaption for PP provided the best results best results among the indirect measurement methods of the influence of gravitational force (AUC = 0.844; Fig. 8).

### Association lesion characteristics and technical parameters with the occurrence of pneumothorax

Spearman correlation showed a high correlation among EGF measurements. Consequently, based on their effect sizes, we included only the RH at EP with adaption for PP in the pneumothorax logistic regression model. Binomial logistic regression analysis showed that RH at EP with adaption for PP (OR 110, 95% CI 9.787–1239.57, *p* < 0.01) and that absence administration of fluid to the pleura (OR 0.106, 95% CI 0.019–0.594, *P* < 0.01) were independently associated with a higher incidence of pneumothorax (Table 2). A strong model fit with an *R*^2^ = 0.555, *p* < *0.01*. Cohen’s f^2^ was 1.25, corresponding to a strong effect [54]. For thoroughness, we conducted binomial logistic regression analyses for the other highly correlated variables; however, each of these models demonstrated a significantly poorer fit compared to the primary model.

**Table 2:** Binomial logistic regression predicting likelihood of pneumothorax

Variable	B	S.E	Wald test	*df*	*P* Value	Odds ratio	95% CI	
							−	+
RH at EP with adaption for PP	4.702	1.235	14.492	1	< 0.001*	110.144	9.787	1239.574
Pleural fluid application	− 2.243	0.879	6.515	1	0.011*	0.106	0.019	0.594
Distance SP (mm)	− 0.018	0.016	1.3	1	0.254	0.982	0.951	1.013
Needle size	1.11	0.723	2.36	1	0.124	3.034	0.736	12.505
Biopsy system	0.44	0.679	0.421	1	0.516	1.553	0.411	5.873

The total number of cases in the cohort was *n* = 128.

B, regression coefficient; S.E., standard error; *df*, degree of freedom; CI, confidence interval; mm, millimeter; RH, relative height; PP, prone position; SP, skin-to-pleura.

## Discussion

**Fig. 9:**
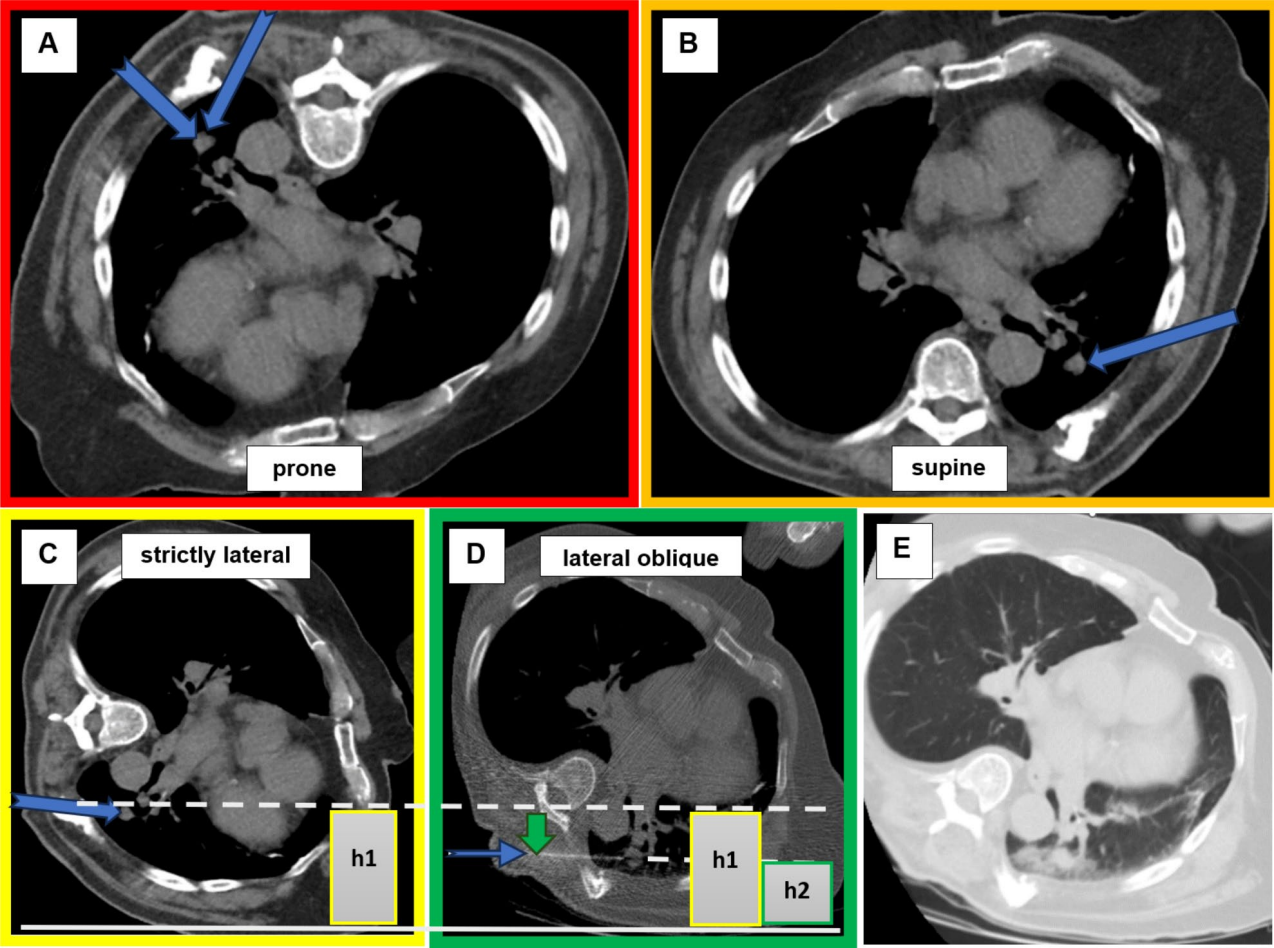
Optimal patient positioning to benefit from the gravitational force of the lungs’ own weight. Color gradation from red to green with green as the best choice for patient positioning. The nearest access routes are shown with a blue arrow. The dashed line represents the height of the entry point, where the biopsy needle passes through the pleura. H1 and H2 illustrate the height difference of the entry point relative to the patient’s baseline in different positions, demonstrating the extent of change caused by repositioning. **A** The reduced decrease in pleural pressure in the prone position, combined with the puncture direction aligned with gravity, significantly increases the risk of pneumothorax; **B, C** In both the supine and lateral positions, the gravitational force of the lung above the access route is utilized. In the strict lateral position, the gravitational force of the mediastinal masses further enhances this effect. **D** The patient is positioned in a lateral oblique position using a wedge cushion, which helps reduce the height of the access route. The green arrow shows the reduced height of the entry point in direct comparison to the strict lateral position. **E** No pneumothorax resulted after the biopsy

Our study found a highly significant and robust correlation between the relative height of the coaxial needle at the entry point, adjusted for the prone position during lung biopsy, and the occurrence of pneumothorax. Increasing the entry point from 0 to 100% of the total lung height was associated with up to a 110-fold increase in the relative risk of pneumothorax. Specifically, raising the entry point from 20 to 50% resulted in approximately a fourfold increase in the relative risk of pneumothorax (*p* < 0.01). Additionally, this method showed the best performance among the indirect measurement methods for assessing the influence of gravity, with an AUC of 0.844. Furthermore, administering fluid into the access route or pleural cavity before lung puncture significantly reduced the relative risk of pneumothorax by ninefold (*p* = 0.011). Both procedures maintained their independent association with a lower incidence of pneumothorax when corrected for other risk factors. This finding is crucial because it allows for strategic patient positioning, where the entry point is placed at the lowest possible position, rather than the lesion, to leverage gravitational force effectively. Ideally, this should be achieved in a lateral position, both to enhance the gravitational force and improve accessibility to the target area (Fig. 9). By utilizing both our easy-to-use calculation method as an indirect measure of the gravitational influence on pleural pressure and artificially increasing pleural pressure before the biopsy through fluid administration near the pleura, the most common complication of CT-guided lung biopsies can be significantly reduced.

Our findings confirm and extend previous research. In dogs, placing them in the biopsy-side down position halted pneumothorax progression [55]. Similarly, Cassel et al. [8] showed a reduced pneumothorax rate by positioning patients (*n* = 80) on the puncture side immediately after the procedure. Drumm et al. [31], followed by Najafi et al. [32], demonstrated that positioning patients in the ipsilateral decubitus position before puncture, as part of the PEARL protocol, also reduced pneumothorax rates. Zidulka et al. [55] suggested that this position decreases the pressure difference between the alveoli and pleura and reduces alveolar size, minimizing pneumothorax occurrence. However, some studies found no reduction in pneumothorax rates with ipsilateral-dependent positioning during biopsy [9, 22]. likely due to the needle path through dependent lung regions rather than the position itself. Our findings reinforce this concept, demonstrating that lung gravity generates a substantial vertical pressure gradient within the pleural cavity, which plays a key role in pneumothorax formation. This supports the findings of West and Matthews [56] and others [57–59], who observed that lung deformation under its own weight and gravitational gradients in pulmonary ventilation and alveolar size affect lung function [47, 60, 61]. Hubmayr et al.[46] first demonstrated this gradient in a supine dog model, revealing both cephalocaudal and vertical gradients. Later, Lai et al.[39, 45] found that pleural pressure also varies within each isogravitational plane. Our univariate analysis and the performance metrics of the GF parameters suggest that a smaller gradient may be present during lung biopsy in the prone position. However, our observed differences between measuring methods were modest, likely due to the limited number of patients in the prone group (*n* = 35). This is consistent with the findings of Nyrén et al., which showed that lung perfusion was more uniformly distributed in the prone position compared to the supine position [62]. However, the wide confidence interval for this variable indicates that additional data and more robust studies are needed to draw definitive conclusions. Although our findings suggest lateral positioning as the preferred approach, its universal applicability should be carefully considered, as not all patients are able to tolerate this position due to underlying medical conditions or physical limitations. Additionally, initially determining the optimal relative entry height may increase both procedure time and patient radiation dose. The practical feasibility of integrating relative height (RH) calculations into clinical workflows also warrants further investigation, particularly with regard to the availability of necessary tools (e.g., existing positioning aids) and the training required for real-time implementation. Future research should focus on strategies to reconcile the theoretical benefits with real-world constraints.

The risk reduction achieved through prior fluid administration to the pleura, as demonstrated in our earlier study [34], suggests that artificially increasing pleural pressure effectively lowers the risk of pneumothorax. This finding is further supported by other research using post-biopsy saline, gelatin sponges, or autologous blood clot seals [14, 28, 30, 63]. Although the number of these specific cases in this expanded cohort was consistent with our earlier study [34], it was important to include this strong predictor in our statistical analysis. It is important to note that fluid administration must be performed by trained hands to avoid the risk of pneumothorax caused by the maneuver itself. However, if fluid or similar substances are administered along the access route after the biopsy, this risk does not exist.

Our reported pneumothorax rate of 57.8% is on the higher end compared to current literature (8–69%, 1–15]). However, this figure should not be interpreted as indicative of selection bias, as we excluded biopsies with potentially protective factors against pneumothorax, such as pleural infiltration (*n* = 44) and significant pleural effusion (*n* = 5). We initially found a significantly higher incidence of pneumothorax with the use of larger biopsy needles (18G vs. 20G, *p* < 0.001) and the side-cut system (48 vs. 25/74; *p* < 0.001). However, this association did not persist when analyzed with other significant variables in the binomial logistic regression. We attribute this discrepancy to a confounding factor -namely, the fact that 20G side-cut biopsy systems are simply not used or available. In the binomial logistic regression, we could not demonstrate an independent association between a shorter skin-to-pleura distance (DSP) and the occurrence of pneumothorax, despite promising results from the univariate analysis. We believe that DSP is indirectly linked to the patient’s physiognomy. Since pleural pressure is significantly influenced by thoracic shape and lung deformation from adjacent structures such as the heart and abdomen [46, 64], we believe there is substantial potential in further exploring this aspect. This also highlights that our model is inherently a simplification.

Consequently, our study has several limitations. First, our measurement models did not account for the exact shape of the thorax and the changes in the chest wall due to breathing during the biopsy. These omissions could explain the underperformance of more physically and theoretically appropriate measurement methods. Second, while obvious pathological changes in the lung parenchyma that significantly impact pleural space physiology [4] were excluded, it must be assumed that early emphysematous or fibrotic changes went undetected by our visual assessment. These exclusions further limit the generalizability of our findings to everyday clinical practice, leaving the extent to which pathological conditions influence physiological processes uncertain. Third, as a retrospective analysis conducted at a single center with a relatively small sample size, this study may further restrict the applicability of its findings. Finally, procedural variability must be considered, as the biopsies were performed by four different interventional radiologists (IRs). Differences in operator technique could introduce variability in the procedure itself and potentially influence the observed complication rates. Given these limitations, our results should be validated through external studies with larger cohorts.

## Conclusion

Enhancing patient positioning by strategically using gravitational force, such as placing the entry point at the lowest possible position, ideally positioning the patient laterally, and applying pre-puncture fluid administration to the pleura could be crucial in reducing pneumothorax during CT-guided lung biopsies.
